# Treatment Outcomes in Patients Receiving Carbon-Ion Radiotherapy Versus Hepatectomy for Hepatocellular Carcinoma (≥4 cm): A Retrospective Study in Japan

**DOI:** 10.3390/jcm14165678

**Published:** 2025-08-11

**Authors:** Keita Maki, Hiroaki Haga, Tomohiro Katsumi, Kyoko Hoshikawa, Takashi Kaneko, Ryosuke Takahashi, Shuichiro Sugawara, Masashi Koto, Fuyuhiko Motoi, Yoshiyuki Ueno

**Affiliations:** 1 Department of Gastroenterology, Yamagata University Faculty of Medicine, Yamagata 990-9585, Japan; k-maki@med.id.yamagata-u.ac.jp (K.M.); y-ueno@med.id.yamagata-u.ac.jp (Y.U.); 2 Department of Radiology, Division of Radiation Oncology, Yamagata University Faculty of Medicine, Yamagata 990-9585, Japan; 3 Department of Surgery I, Yamagata University Graduate School of Medical Science, Yamagata 990-9585, Japan

**Keywords:** carbon-ion radiotherapy, hepatectomy, hepatocellular carcinoma, intrahepatic distant control rate, local control rate

## Abstract

**Background/Objectives**: Carbon-ion radiotherapy (CIRT) is now covered by Japan’s health insurance for patients with hepatocellular carcinoma (HCC) tumors measuring ≥4 cm. However, no studies have compared intrahepatic control between CIRT and hepatectomy in these patients. **Methods**: We retrospectively analyzed intrahepatic control in 51 patients with HCC tumors ≥4 cm. Among them, 38 underwent CIRT (60 Gy in four fractions), while 13 underwent systematic hepatectomy. Intrahepatic recurrence was classified as local or intrahepatic distant. We evaluated local, distant, and total intrahepatic control rates at 1 and 2 years. **Results**: In the CIRT group, the local control rates at 1 and 2 years were 81.5% and 76.3%, whereas the intrahepatic distant control rates were 68.5% and 63.2% (*p* = 0.0495), respectively. Among patients aged <80 years, the 2-year intrahepatic control rate did not significantly differ between CIRT and hepatectomy. However, it was significantly lower in patients aged ≥80 years treated with CIRT than in those aged <80 years (73.7% vs. 42.1%, *p* = 0.0100), with similar trends in local (92.3% vs. 63.2%, *p* = 0.0381) and distant control (78.9% vs. 47.3%, *p* = 0.0259). **Conclusions**: CIRT may be as effective as hepatectomy for HCC tumors ≥4 cm in patients aged <80 years, but its efficacy declines in older patients, warranting age-tailored strategies.

## 1. Introduction

Globally, hepatocellular carcinoma (HCC) is the sixth most common malignancy and the third leading cause of cancer-related mortality. HCC frequently develops in patients with chronic liver disease [1]. Its treatment options include surgical resection, radiofrequency ablation (RFA), transarterial chemoembolization (TACE), and radiation therapy, with surgery and RFA being the most effective. Since 2022, particle beam therapy has been covered by Japan’s health insurance for HCC tumors measuring ≥4 cm that are difficult to resect surgically, broadening treatment options for liver cancer. Particle beam therapies such as carbon-ion radiotherapy (CIRT) and proton beam therapy have demonstrated high efficacy and safety profiles, thereby garnering increasing attention.

Compared with proton or X-ray therapy, CIRT has a higher linear energy transfer and greater relative biological effectiveness [2]. The Bragg peak effect allows precise dose delivery to the tumor while minimizing exposure to surrounding healthy tissues [3]. In addition, CIRT requires fewer treatment sessions, thereby reducing treatment burden and preserving patients’ quality of life [3].

The outcomes of CIRT irradiation have been recently reported in patients treated at various medical facilities. For example, CIRT can achieve a high cure rate. In multiple prospective and multicenter studies, CIRT has demonstrated high local control rates and low toxicity rates in patients with HCC [4,5,6,7,8,9,10,11,12,13,14,15,16,17].

Currently, CIRT is covered by Japan’s health insurance for patients with HCC measuring ≥4 cm that cannot be treated curatively, such as by hepatectomy. Owing to its reduced physical burden, CIRT is increasingly considered for older patients with HCC.

While many studies have reported on the local control of CIRT at the irradiated site, few have investigated intrahepatic distant recurrence. Presently, no studies have examined the intrahepatic control rate following CIRT specifically for tumors measuring ≥4 cm that are covered by Japan’s health insurance.

For HCC ≥4 cm, both surgical resection and particle beam therapy, including CIRT, are considered effective treatment options with high cure rates. However, no study has directly compared intrahepatic control rates between CIRT and hepatectomy for such tumors. Hence, this study aimed to focus on examining HCC tumors ≥4 cm that meet Japan’s current insurance coverage criteria. To our knowledge, this research is the first to report on this topic (Supplementary Figure S1). The results of this study may provide new insights that are useful for clinical decision-making, particularly in choosing between CIRT and hepatectomy, for patients with HCC ≥4 cm.

## 2. Materials and Methods

### 2.1. Patient Population

This study included 86 patients diagnosed with HCC tumors measuring ≥4 cm at our hospital between November 2022 and December 2023 (Supplementary Figure S2). However, 35 were excluded for the following reasons: systemic therapy (*n* = 11), chemotherapy after CIRT because of positive vascular invasion (*n* = 2), nonsingle CIRT irradiation caused by stage 2 irradiation (*n* = 9), follow-up period <3 months (*n* = 3), duplicate cancer (*n* = 1), treatment with transarterial embolization after rupture (*n* = 5), or best supportive care policy (*n* = 4). Consequently, 38 patients who received CIRT for HCC tumors ≥4 cm were ultimately included in the analysis.

Additionally, we included 13 patients who underwent systematic hepatectomy for HCC tumors ≥4 cm at our hospital between January 2019 and December 2023. Segmental liver resection based on tumor-bearing portal tributaries defined systematic hepatectomy. Patients with extrahepatic spread were excluded.

HCC diagnosis, vascular invasion, lymph node involvement, and distant metastasis were assessed via contrast-enhanced computed tomography (CT) and/or magnetic resonance imaging (MRI). If these imaging methods could not confirm HCC, the tumors were diagnosed histologically via tumor biopsy.

Patients diagnosed with HCC with tumors ≥4 cm, from January 2019 to October 2022, underwent surgical resection in accordance with Makuuchi’s criteria [18]. CIRT became available under insurance coverage at our institution from November 2022. Patients diagnosed between November 2022 and December 2023 were also treated surgically in accordance with Makuuchi’s criteria. However, CIRT was proposed as an alternative for some patients, such as those with a high risk of complications or those who preferred less invasive treatments. In each case, the treatment strategy was discussed in a multidisciplinary conference, and the final decision was made after obtaining informed consent from the patient.

### 2.2. Carbon-Ion Radiotherapy

The spot scanning method and a rotating-gantry beam system were employed, enabling 360° irradiation from any angle. Before treatment planning, fiducial metallic markers were implanted near the tumor to ensure precise positioning during CIRT. To maintain target reproducibility, clinicians used a low-temperature thermoplastic sheet, a vacuum bag, and a respiratory-gated irradiation system for CT-based radiotherapy planning. Gross tumor volume (GTV) was determined using dynamic contrast-enhanced CT and/or MRI, whereas clinical target volume (CTV) encompassed a 7 mm margin from the GTV. Treatment planning was conducted using a CT-based three-dimensional system with RayStation (RaySearch Laboratories, Stockholm, Sweden), optimized using tools for robustness. Setup errors were compensated for with a 2 mm margin in left–right, anterior–posterior, and superior–inferior directions. A software setting of 2% was employed for the range uncertainty parameter.

The CT scan for treatment planning was taken using a four-dimensional CT scan synchronized with respiratory movements. Then, the CT images were divided into 10 respiratory phases in 10% increments from 0% to 100%, with the inhalation and exhalation phases corresponding to 0% and 50%, respectively. The tumor was contoured on the 50% (exhalation) phase image. The internal target volume (ITV) was then set by combining the CT images with the maximum phase range where the GTV movement was within 3 mm. Only the phases within that range were used during actual irradiation.

Before each treatment, orthogonal fluoroscopy and radiography confirmed and corrected the radiation field as needed. The prescribed dose of CIRT was expressed in Gy, calculated as the absorbed dose multiplied by the relative biological efficacy of carbon ions. According to the institutional protocol, the dose for HCC was 60 Gy, delivered in 4 fractions (4 Fr). This four-fraction treatment was administered once per day for 4 consecutive days. For organ at risk (OAR) constraints, we set the remnant liver volume receiving less than 30 Gy for the liver parenchyma to be at least 500 cc. The dose to the most exposed 2 cc (D2cc) was also set to be less than 30 Gy for the digestive tract.

### 2.3. Clinical Outcome Evaluation

Patients were assessed at 1 month after CIRT and then every 3 months for the first 2 years, every 6 months for the following 2 years, and annually thereafter. Follow-up evaluations included physical examinations, laboratory tests, and dynamic contrast-enhanced CT or MRI. The final follow-up date was in January 2025. For post-hepatectomy follow-up, a similar protocol was applied.

For CIRT, we defined local recurrence as radiographic findings of tumor enlargement or new early-enhanced areas within the treatment field, where contrast enhancement had disappeared after CIRT, according to the RECIST v1.1 guidelines. Recurrence outside the treated field within the liver indicated intrahepatic distant recurrence (Supplementary Figure S1).

For systematic hepatectomy, we defined local recurrence as new early-enhanced areas on the resection surface as per the RECIST v1.1 guidelines. Recurrence within the liver but not on the resection surface defined intrahepatic distant recurrence (Supplementary Figure S1).

Moreover, progression-free survival (PFS) was measured from the date of initial CIRT or systematic hepatectomy to the date of local or distant intrahepatic recurrence or death from any cause. The overall survival (OS) was calculated from the date of initiation of CIRT or systematic hepatectomy to the date of death from any cause.

Tumor response was assessed using the RECIST v1.1 and mRECIST guidelines, with a consensus among radiologists. In line with RECIST v1.1, responses were evaluated according to the sum of the longest diameters of all lesions. The mRECIST criteria defined complete response (CR) as enhancement disappearance in all lesions, partial response (PR) as a ≥30% reduction in the sum of viable enhanced lesion diameters, progressive disease (PD) as a ≥20% increase, and stable disease as a response between PR and PD. The maximum response recorded before local progression was considered as the best treatment response.

Assessment of CIRT-associated toxicity was based on the Common Terminology Criteria for Adverse Events (CTCAE), version 5.0. CTCAE classifies the severity of adverse events (AEs) using a grading system from 1 to 5, where 1 indicates mild, 2 indicates moderate, 3 indicates severe, 4 indicates life-threatening, and 5 indicates death. Tumor progression and symptoms attributed to the tumor or other treatments for recurrent tumors were not taken into consideration while assessing CIRT-related toxicity. Assessments included physical examinations (skin condition, digestive and respiratory symptoms, pleural and abdominal effusion, and jaundice), blood tests (levels of total bilirubin, albumin, aspartate aminotransferase, and alanine aminotransferase; platelet count; prothrombin time; prothrombin time–international normalized ratio; alpha-fetoprotein levels; and protein-induced vitamin K absence or antagonist-II levels), as well as imaging tests (contrast-enhanced CT or MRI) at 3, 6, 9, 12, 15, 18, 21, and 24 months post treatment. AEs occurring within 90 days of CIRT initiation were defined as early AEs, whereas those occurring after 90 days were defined as late AEs.

### 2.4. Statistical Analysis

Local recurrence rates, intrahepatic distant recurrence rates, PFS, and OS were analyzed using the Kaplan–Meier method. Using the log-rank test, we compared these parameters between the CIRT and hepatectomy groups. These groups, as well as the recurrence and nonrecurrence groups following CIRT, were compared using the *t*-test for continuous variables and the χ^2^ test or the Fisher’s exact test for categorical variables. Given the small sample size, we conducted the Shapiro–Wilk test before the *t*-test to confirm data normality. These statistical data were analyzed using GraphPad Prism-9. Matching was performed using a 1:1 nearest-neighbor algorithm with replacement in the FNN package in R. This enabled the surgery patients to be matched to more than one patient undergoing CIRT. Therefore, some surgery patients were included multiple times in the matched dataset. After matching, Fisher’s exact test was used for comparing the groups. All statistical data were analyzed with a significance threshold of *p* < 0.05.

### 2.5. Ethics Approval

The Ethics Review Committee of Yamagata University School of Medicine approved this retrospective study (Approval No.: 2023-110).

## 3. Results

### 3.1. Characteristics of Patients Who Underwent CIRT

This study included 29 males and 9 females (median [range] age: 80 (56–93) years) who underwent CIRT. Regarding modified albumin–bilirubin (mALBI) grades, 20, 9, 8, and 3 patients were graded as 1, 2a, 2b, and 3, respectively, with a median ALBI score of −2.633 (−3.415 to −1.313). In terms of the Barcelona Clinic Liver Cancer (BCLC) staging system, 30, 3, and 5 patients were classified as stages A, B, and C, respectively.

Among the 38 included patients, 26 received CIRT as their initial treatment, whereas the remaining 12 had prior treatments, including surgery (*n* = 5), RFA (*n* = 4), TACE (*n* = 7), and systemic therapy (atezolizumab + bevacizumab, *n* = 1; lenvatinib, *n* = 2). The median maximum tumor diameter was 53.5 (40–110) mm. Additionally, 35 patients had a single liver tumor, while 3 had two tumors. Microvascular invasion was absent in 32 patients and present in 6 patients (Table 1).

### 3.2. Recurrence/Nonrecurrence After CIRT

The median follow-up period was 660 (143–901) days. No patient died during the study period. Of the 38 patients, 17 developed recurrence, with local recurrence, distant intrahepatic recurrence, and both observed in 9, 14, and 6 patients, respectively. The recurrence group was significantly older than the nonrecurrence group (84 (67–93) vs. 75 (56–91) years; *p* = 0.0380) (Table 1).

**Figure 1:**
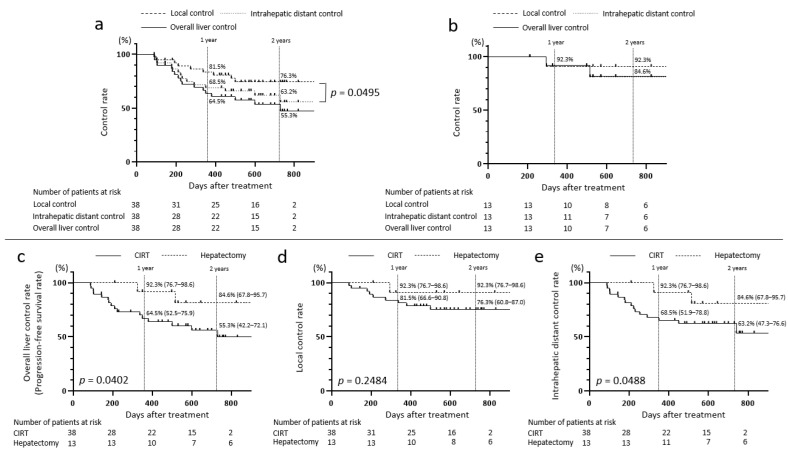
Intrahepatic control rates after CIRT and hepatectomy for HCC tumors measuring ≥4 cm. Overall liver control rate, local control rate, and intrahepatic distant control rate after (**a**) CIRT and (**b**) hepatectomy. (**c**) Progression-free survival rates, (**d**) local control rates, and (**e**) intrahepatic distant control rates for the CIRT and hepatectomy groups. CIRT, carbon-ion radiotherapy.

The overall 1- and 2-year liver control rates (PFS) after CIRT for HCC tumors ≥4 cm were 64.5% and 55.3%, respectively (Figure 1a). At 1 and 2 years, the intrahepatic distant control rates were 68.5% and 63.2%, which were lower than the local control rates (81.5% and 76.3%, respectively) (*p* = 0.0495; Figure 1a).

### 3.3. Characteristics of Patients Who Underwent Hepatectomy

Among the patients who underwent hepatectomy, 11 were males and 2 were females (median age: 70 (58–77) years). Regarding the mALBI grades, 10, 2, 1, and 1 patients were graded as 1, 2a, 2b, and 3, respectively, with a median ALBI score of −2.786 (−3.186 to −1.800). In terms of tumor stage, 8, 4, and 1 patients were classified as BCLC Stage A, B, and C, respectively.

Eight patients underwent hepatectomy as their initial treatment, whereas five had received prior treatment, including surgery, RFA, TACE, or systemic therapy. The median maximum tumor diameter was 50.0 (40–130) mm. Furthermore, nine patients had a single liver tumor, whereas four had two tumors. Microvascular invasion was absent in twelve patients and present in one.

The CIRT group was significantly older than the hepatectomy group (80 (56–93) years; *p* = 0.0046). This group also had a significantly higher proportion of patients with grade 2a/2b mALBI (*p* = 0.045; Table 2).

### 3.4. Disease Control Rates and OS Rates in the CIRT and Hepatectomy Groups

The median follow-up period for patients undergoing hepatectomy was 800 (210–2278) days. No patient died during the study period. Of the 13 patients who underwent hepatectomy, two experienced recurrence, with one experiencing local recurrence and the other experiencing distant intrahepatic recurrence.

The 1- and 2-year liver control rates (PFS rates) following hepatectomy for HCC tumors measuring ≥4 cm were 92.3% and 84.6%, respectively (Figure 1b). At 1 and 2 years after hepatectomy, the local control rates were 92.3% and 92.3%, whereas the intrahepatic distant control rates were 92.3% and 84.6%, respectively (Figure 1b).

Between the two groups, the overall 1- and 2-year liver control rates (PFS rates) were significantly lower in the CIRT group than in the hepatectomy group (at 1 year: 64.5% (95% CI: 52.5–75.9%) vs. 92.3% (95% CI: 76.7–98.6%); at 2 years: 55.3% (95% CI: 42.2–72.1%) vs. 84.6% (95% CI: 67.8–95.7%), *p* = 0.0402; Figure 1c). The local control rates were also lower in the CIRT group at 1 (81.5% (95% CI: 66.6–90.8%) vs. 92.3% (95% CI: 76.7–98.6%)) and 2 (76.3% (95% CI: 60.8–87.0%) vs. 92.3% (95% CI: 76.7–98.6%)) years (Figure 1d). Similarly, intrahepatic distant control rates were lower in the CIRT group at 1 (68.5% (95% CI: 51.9–78.8%) vs. 92.3% (95% CI: 76.7–98.6%)) and 2 (63.2% (95% CI: 47.3–76.6%) vs. 84.6% (95% CI: 67.8–95.7%)) years (*p* = 0.0488; Figure 1e). Importantly, no patients in either treatment group died within the observation period, precluding the reporting of OS rates.

The 1- and 2-year OS rates in the CIRT group were 97.4% and 94.7%, respectively, whereas those in the hepatectomy group were 100% and 100%, respectively. No significant difference was observed between the two groups (*p* = 0.3974). During the follow-up period, two patients in the CIRT group died because of disease progression (*n* = 1) and cholangitis (*n* = 1).

### 3.5. Comparison Between the CIRT and Hepatectomy Groups in Patients Aged Below 80 Years

**Figure 2:**
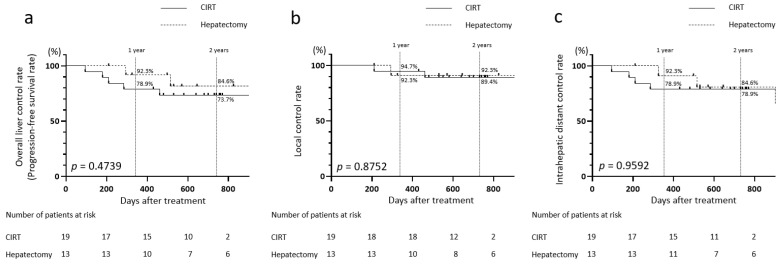
Intrahepatic control rates after CIRT and hepatectomy in patients aged <80 years with HCC tumors measuring ≥4 cm. (**a**) Progression-free survival rates, (**b**) local control rates, and (**c**) intrahepatic distant control rates for the CIRT and hepatectomy groups.

Given that all patients in the hepatectomy group were below 80 years old, we standardized the age factors to compare patients aged below 80 years in the CIRT and hepatectomy groups (Table 3). The overall 1- and 2-year liver control rates (PFS rates) were lower after CIRT than after hepatectomy (at 1 year: 78.9% vs. 92.3%; at 2 years: 73.7% vs. 84.6%, Figure 2a). However, the CIRT group had a higher 1-year local control rate than the hepatectomy group (94.7% vs. 92.3%) but obtained a lower 2-year rate (89.4% vs. 92.3%; Figure 2b). Additionally, the 1- and 2-year intrahepatic distant control rates were lower after CIRT than after hepatectomy (at 1 year: 78.9% vs. 92.3%; at 2 years: 78.9% vs. 84.6%, Figure 2c).

However, these findings indicate no significant differences between the two groups aged below 80 years in terms of overall liver control rates, local control rates, and intrahepatic distant control rates.

### 3.6. Comparison Between the CIRT and Hepatectomy Groups Using Matching

Age and mALBI grade were used as covariates to perform 1:1 nearest-neighbor matching. Matching of each patient in the CIRT group (*n* = 38) to the closest patient in the surgery group was based on the Euclidean distance in the two-dimensional space of age and mALBI grade. Matching was performed with replacement, enabling a single surgery patient to be selected for multiple CIRT patients, if optimal. After matching, both groups consisted of 38 patients. PFS rates were compared between the CIRT and surgery groups after matching for age and mALBI grade. The 1- and 2-year PFS rates in the CIRT group were 64.5% and 55.3%, respectively, whereas those in the hepatectomy group were 86.8% and 64.5%, respectively (Supplementary Figure S3). No significant difference was observed between the two groups (*p* = 0.6200).

### 3.7. Comparison of Disease Control Rates in Patients with CIRT Aged Below 80 Years and 80 Years and Older

**Figure 3:**
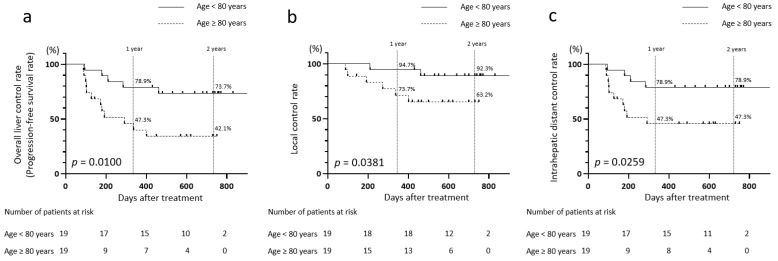
Intrahepatic control rates in patients aged <80 years versus ≥80 years who underwent CIRT for HCC tumors measuring ≥4 cm. (**a**) PFS rates, (**b**) local control rates, and (**c**) intrahepatic distant control rates in the <80- and ≥80-year CIRT groups.

Patients aged <80 and ≥80 years who underwent CIRT were compared (Table 4). The overall 1- and 2-year liver control rates (PFS rates) were higher in patients aged <80 years than those aged ≥80 years (at 1 year: 78.9% vs. 47.3%; at 2 years: 73.7% vs. 42.1%) (*p* = 0.0100; Figure 3a). Likewise, both the 1- and 2-year local control rates were higher in patients aged <80 years than in those aged ≥80 years (at 1 year: 94.7% vs. 73.7%; at 2 years: 92.3% vs. 63.2%) (*p* = 0.0381; Figure 3b). The 1- and 2-year intrahepatic distant control rates also showed similar results, with 78.9% and 47.3% obtained in both age groups, respectively (*p* = 0.0259; Figure 3c).

These results demonstrate that the overall liver control rates, local control rates, and intrahepatic distant control rates significantly differed between the two age groups who underwent CIRT.

### 3.8. AEs After CIRT

The treatment course was successfully completed by all 38 patients without any interruptions. Figure 1 depicts the incidence and onset of AEs observed during the follow-up period. The most common AEs observed were liver dysfunction (*n* = 13, 34.2%), fatigue (*n* = 12, 31.6%), and skin redness/dermatitis (*n* = 11, 28.9%). Most events were classified as grade 1 or 2. Grade 3 AEs were rare and were observed in only one patient (3.9%) with biliary stricture. The patient was treated with endoscopic retrograde cholangiopancreatography and stenting, which improved the symptoms, and the patient was discharged on the 10th day of hospitalization. Early AEs (onset within 3 months) included skin redness/dermatitis (median: 1.5 months), anorexia (median: 1.5 months), fatigue (median: 2.0 months), liver dysfunction (median: 2.2 months), itchy skin (median: 2.5 months), and nausea (median: 3.0 months). Late AEs (onset after 3 months) included localized pleural effusion (median: 4.5 months), biliary stricture (median: 5.0 months), and rib fracture/myositis (median: 6.0 months) (Table 5).

## 4. Discussion

This study is the first to report intrahepatic control outcomes after CIRT in patients with HCC measuring ≥4 cm and the first to compare these outcomes with those after undergoing surgical resection in the same tumor size category. The findings of this study provide new insights into the therapeutic potential of CIRT, particularly in patients in whom standard curative approaches such as hepatectomy may not be feasible.

In this study, we classified intrahepatic recurrence as either local recurrence (confined to the irradiated region) or intrahepatic distant recurrence (occurring outside the irradiated region). We retrospectively assessed the intrahepatic control, local control, and intrahepatic distant control rates at 1 and 2 years following CIRT. Patients treated with CIRT were compared with those who underwent hepatectomy. Furthermore, subgroup analyses were conducted to evaluate the impact of age on treatment outcomes. In particular, we compared patients aged <80 years who underwent hepatectomy with those who received CIRT, as well as CIRT-treated patients aged <80 years with those aged ≥80 years. To reduce potential confounding factors such as age and mALBI grade, we additionally conducted 1:1 nearest-neighbor matching.

Our findings demonstrated the following: (1) the intrahepatic distant control rate was lower than the local control rate following CIRT for HCC tumors ≥4 cm; (2) the intrahepatic control rates did not significantly differ between CIRT and hepatectomy in patients aged <80 years; and (3) the intrahepatic control rate after CIRT was significantly lower in patients aged ≥80 years than in those aged <80 years. Therefore, age may be an important factor influencing intrahepatic disease control after CIRT. In particular, patients aged ≥80 years may be more at risk of developing intrahepatic recurrence following CIRT for HCC ≥4 cm.

Currently, both Japanese and Western liver cancer treatment guidelines consider surgical resection as the most effective curative option for solitary HCC tumors measuring ≥4 cm [19,20]. However, this treatment option is not feasible for some patients because of advanced age or underlying comorbidities.

Nonsurgical treatments such as TACE and RFA are widely used for treating HCC in addition to surgical resection and radiotherapy. However, the efficacy of TACE reduces with increasing tumor size, leading to increased local recurrence rates resulting from incomplete embolization [21,22]. RFA is also associated with technical limitations for tumors >3 cm, particularly when anatomical location restricts adequate ablation [23]. In this context, CIRT has emerged as a promising nonsurgical option, offering high treatment precision and being applicable for treating larger tumors.

CIRT is indicated for patients with HCC tumors ≥4 cm who are unsuitable candidates for conventional curative therapies and who have preserved liver function classified as Child–Pugh class A or B. Notably, this treatment method can be offered to patients deemed ineligible for surgical resection. In addition, CIRT can be performed on an outpatient basis, thereby imposing less physical stress on patients. Although CIRT dosing and fractionation schedules vary among institutions, our facility administers 60 Gy in four fractions. Given the increased perioperative risks in older patients, CIRT may be a promising alternative to surgical resection for this population.

Various clinical outcomes of CIRT for HCC have already been reported. A PubMed search conducted for the 2017–2023 period using the keywords “hepatocellular carcinoma,” “carbon-ion radiotherapy,” and “heavy-ion radiotherapy” identified 15 relevant studies, including our own (13 from Japan and 2 from China; Supplementary Table S1). Reported 1- and 3-year local control rates following CIRT were 93.4–100% and 76.5–94.4%, respectively. In the two Chinese studies by Hong et al. [16] and Zhang et al. [17], TACE was strongly recommended before and/or after CIRT, with 72.2% of patients in Zhang et al. receiving one to three TACE cycles before treatment. Conversely, all Japanese studies, including ours, administered heavy-ion radiotherapy alone. Furthermore, the median patient ages in the Chinese studies were 57 (28–76) and 58.5 (28–87) years, respectively [16,17], whereas the Japanese studies generally included older populations, with median ages in the 70s to 80s. These differences should be considered when interpreting the results.

In our study, the 1- and 2-year local control rates following CIRT were 81.5% and 76.3%, respectively, which are lower than those reported in previous studies (Supplementary Table S1). This discrepancy may be explained by two primary factors. First, this study was the only one specifically limited to large HCCs (≥4 cm), a category eligible for insurance coverage in Japan. Second, the median patient age was 80 years, which was higher than that in other studies. Although Shiba et al. [5] also included older adults (median age: 83 years), the median tumor size in their study was smaller (45 (15–93) mm) than that in ours. Differences in patient backgrounds, including tumor size, patient age, and TACE use and timing in relation to CIRT, may have had a substantial impact on treatment outcomes. To our knowledge, this study is the first to report hepatic control rates in a cohort with a median age of 80 years and tumor sizes ≥4 cm, which aligns with the criteria for Japan’s health insurance reimbursement.

Moreover, the 1- and 2-year local control rates following CIRT were 81.5% and 76.3%, respectively, and the 1- and 2-year intrahepatic distant control rates were 68.5% and 63.2%, respectively (Figure 1a). These findings suggest that achieving disease control is more challenging in intrahepatic distant regions than in the irradiated local region, consistent with the results reported by Zhang et al. [17].

The mean age of patients who underwent hepatectomy was 70 years (maximum: 77 years), whereas that of those treated with CIRT was 80 years (maximum: 93 years); thus, patients receiving CIRT were significantly older than those undergoing hepatectomy (*p* = 0.0046; Table 2). Additionally, the mALBI grade was significantly lower in the CIRT group than in the hepatectomy group (*p* = 0.045; Table 2). The modified ALBI (mALBI) score is a refinement of the ALBI score for liver function and has been reported to be a useful prognostic indicator after treatments such as hepatic resection [24], stereotactic body radiotherapy [25], and CIRT [12]. Older patients with HCC and reduced hepatic reserve are more likely to select less invasive treatment options; hence, the preference for CIRT in this cohort is understandable. In this study, the majority of adverse events following CIRT were grade 1–2, indicating that the procedure may be relatively safe even in elderly patients.

The 2-year intrahepatic distant control rate was significantly higher in the hepatectomy group than in the CIRT group (84.6% vs. 63.2%, *p* = 0.0488). The overall 2-year intrahepatic control rate was also significantly higher in the hepatectomy group (84.6% vs. 55.3%, *p* = 0.0402). However, considering the baseline differences in age and hepatic function between the two groups, these results should be interpreted with caution.

The overall 1- and 2-year liver control rates at our institution (PFS) following liver resection for HCC with a median tumor size of 5.0 cm were 92.3% and 84.6%, respectively. A study conducted by Shehta et al., involving tumors of comparable size, reported 1- and 3-year PFS rates of 74.2% and 38.5%, respectively, for HCCs measuring 5–10 cm (median = 6.0 cm) [26]. Treatment results at each facility should be interpreted with caution, as patient characteristics such as tumor size and the extent of vascular invasion might vary across institutions.

At our hospital, no patients aged >80 years underwent hepatectomy. Therefore, we examined hepatic control outcomes in patients aged <80 years with HCC treated with either CIRT or hepatectomy (Table 3). The 1- and 2-year overall hepatic control rates, local control rates, or intrahepatic distant control rates did not significantly differ between the CIRT and hepatectomy groups. Larger-scale studies are warranted to validate these findings.

Given that the recurrence group following CIRT was significantly older than the nonrecurrence group (*p* = 0.0380; Table 1), we identified “age ≥ 80 years” as a potential risk factor for recurrence. On the basis of this factor, patients were stratified into two age groups: <80 and ≥80 years (Table 4). The overall hepatic control rate (PFS), local control rate, and intrahepatic distant control rate at 1 and 2 years after CIRT were significantly higher in the <80-year group than in the ≥80-year group (Figure 3). Thus, patient age may be a key factor influencing intrahepatic disease control after CIRT.

Cellular senescence of hepatocytes has been suggested to promote malignant transformation into HCC cells and thereby play a central role in hepatocarcinogenesis [27,28]. In addition, age-related immune senescence (immunosenescence) has been reported to contribute to increased cancer risk through impaired antitumor immunity by inducing various alterations across the immune system [29]. These mechanisms might partially explain the reduced intrahepatic control observed in the elderly population in this study.

In stereotactic body radiotherapy for HCC, a previous study reported that while disease-free survival was significantly shorter in the elderly group (≥75 years) than in the younger group (<75 years), no significant differences were observed in local control, OS, or incidence of AEs [30]. Given the increased physiological burden of hepatectomy in older patients, CIRT is a less invasive alternative with potential clinical benefits. However, in patients with HCC tumors ≥4 cm, the risks of local and intrahepatic distant recurrences following CIRT should be carefully considered during treatment planning. Considering the differences in underlying liver disease profiles (e.g., viral hepatitis and MAFLD/MASH), accessibility to CIRT, and surgical risk assessment criteria across countries, the findings of this study, which has been conducted within the Japanese healthcare context, must be interpreted with caution when applied internationally.

This study has several limitations that must be acknowledged. First, it was a retrospective, single-center analysis with a relatively small sample size, thereby limiting the generalizability of the findings and reducing the statistical power. Second, because of the small sample size in the hepatectomy group, the results must be interpreted with caution, considering the potential risk of a Type II error. Third, age and liver function at baseline differed between the CIRT and hepatectomy groups, thereby introducing potential confounding factors that may have influenced the observed outcomes. Owing to the limited sample size, additional matching procedures were employed to minimize potential confounding. However, further investigation is warranted as more cases are reported in future studies. Fourth, randomization was not performed, precluding definitive conclusions regarding treatment superiority. Fifth, patients who had received prior antitumor treatments were included in both the CIRT and liver resection groups, which could have introduced bias into the analysis. Future multicenter prospective studies with matched patient cohorts are warranted to validate our findings and refine CIRT’s role in older patients with large HCC.

To the best of our knowledge, this study is the first to report intrahepatic control rates after CIRT for HCC tumors ≥4 cm, which are difficult to treat using conventional curative treatments such as hepatectomy and are now covered by Japan’s health insurance. Our results indicate that intrahepatic distant control is more challenging than local control after CIRT. In patients aged <80 years, intrahepatic control rates showed no significant difference between CIRT and hepatectomy; thus, the superiority of either treatment was difficult to identify. However, patients aged ≥80 years with HCC tumors ≥4 cm were more at risk for both local and distant recurrences following CIRT; therefore, strategies to mitigate recurrence risks must be developed. Thus, the findings of this study may contribute to the development of improved treatment strategies for liver cancer.

## 5. Conclusions

CIRT provides comparable intrahepatic control to surgical resection for HCC tumors ≥4 cm in patients aged <80 years with preserved liver function. However, in patients aged ≥80 years, intrahepatic control after CIRT was significantly lower, especially in non-irradiated areas. These findings highlight that age must always be considered during treatment planning and support the need for individualized strategies when using CIRT, particularly in older patients.

## Figures and Tables

**Table 1:** Characteristics of patients who underwent CIRT.

	CIRT (*n* = 38)	Recurrence (*n* = 17)	No Recurrence (*n* = 21)	*p* Value
Age	Median: 80 (range: 56–93)	84 (67–93)	75 (56–91)	0.0380
Sex (male/female)	29/9	12/5	17/4	0.1855
Etiology (HBV/HCV/non-B, non-C/alcohol/MASH/PBC)	5/10/7/8/6/2	4/6/0/3/3/1	1/4/7/5/3/1	0.0598
mALBI grade (1/2a/2b/3)	20/9/8/1	8/4/5/0	12/5/3/1	0.6716
ALBI score	Median: −2.633 (−3.415 to −1.313)	−2.596 (−3.075 to −1.744)	−2.688 (−3.415 to −1.313)	0.6873
BCLC stage (A/B/C)	30/3/5	11/2/4	19/1/1	0.1724
First treatment/pretreatment (surgery/RFA/TACE/systemic therapy)	26/12	9/8	17/4	0.0873
Size (mm)	53.5 (40–110)	56.0 (42–101)	50.0 (40–110)	0.4081
Total number of intrahepatic tumors (1/2)	35/3	14/3	21/0	0.1935
MVI (−/+)	32/6	13/4	19/2	0.3776
Blood biochemistry (median [range])				
T-bil level (mg/dL)	0.60 (0.40–2.2)	0.70 (0.40–1.4)	0.60 (0.40–2.2)	0.9175
Alb level (g/dL)	3.9 (2.7–4.9)	3.9 (2.7–4.5)	4.0 (2.7–4.9)	0.7185
AST level (U/L)	32.0 (12–222)	36.0 (12–77)	31.0 (13–222)	0.3799
ALT level (U/L)	27.0 (5.0–135)	26.0 (5–80)	31.0 (9–135)	0.5084
PLT count (10^3^/µL)	176 (45–382)	196.0 (45.0–346.0)	166.0 (55.0–382.0)	0.8904
PT%	94.0 (65–114)	92.0 (65–114)	96.0 (76–112)	0.2655
AFP level (ng/mL)	4.8 (2.0–20,000)	6.7 (2.8–20,000)	4.0 (2.0–17,455)	0.1770
PIVKA-II level (mAU/mL)	203.0 (13–81,886)	552.0 (21–21,680)	140.0 (13.0–81,886)	0.5650

AFP, alpha-fetoprotein; Alb, albumin; ALBI score, albumin–bilirubin score; ALT, alanine transaminase; AST, aspartate transaminase; BCLC, Barcelona Clinic Liver Cancer; CIRT, carbon-ion radiotherapy; HBV, hepatitis B virus; HCV, hepatitis C virus; mALBI grade, modified albumin–bilirubin grade; MASH, metabolic dysfunction-associated steatohepatitis; MVI, macrovascular invasion; PBC, primary biliary cholangitis; PIVKA-II, protein-induced vitamin K absence or antagonist-II; PLT, platelet count; PT, prothrombin time; RFA, radiofrequency ablation; TACE, transarterial chemoembolization; T-bil, total bilirubin.

**Table 2:** Characteristics of patients undergoing hepatectomy and those receiving CIRT.

	CIRT (*n* = 38)	Hepatectomy (*n* = 13)	*p* Value
Age	Median: 80 (range: 56–93)	70 (58–77)	0.0046
Sex (male/female)	29/9	11/2	0.7063
Etiology (HBV/HCV/non-B, non-C/alcohol/MASH/PBC)	5/10/7/8/6/2	1/1/4/6/1/0	0.5837
mALBI grade (1/2a/2b/3)	20/9/8/1	10/2/1/0	0.0450
ALBI score	−2.633 (−3.415 to −1.313)	−2.786 (−3.186 to −1.800)	0.1499
BCLC stage (A/B/C)	30/3/5	8/4/1	0.1403
First treatment/pre-treatment (surgery/RFA/TACE/systemic therapy)	26/12	8/5	0.7458
Size (mm)	53.5 (40–110)	50.0 (40–130)	0.2893
Total number of intrahepatic tumors (1/2)	35/3	9/4	0.0605
MVI (−/+)	32/6	12/1	0.6618
Blood biochemistry (median [range])			
T-bil level (mg/dL)	0.60 (0.40–2.2)	0.70 (0.30–0.90)	0.2348
Alb level (g/dL)	3.9 (2.7–4.9)	4.1 (3.0–4.3)	0.4139
AST level (U/L)	32.0 (12–222)	24.0 (14–77)	0.2399
ALT level (U/L)	27.0 (5.0–135)	22.0 (13–107)	0.6917
PLT count (10^3^/µL)	176 (45–382)	190 (102–252)	0.7654
PT%	94.0 (65–114)	93.0 (68–123)	0.8252
AFP level (ng/mL)	4.8 (2.0–20,000)	4.0(2.0–1273)	0.2729
PIVKA-II level (mAU/mL)	203.0 (13–81,886)	82.0(18.0–7719)	0.4114

AFP, alpha-fetoprotein; Alb, albumin; ALBI score, albumin–bilirubin score; ALT, alanine transaminase; AST, aspartate transaminase; BCLC, Barcelona Clinic Liver Cancer; CIRT, carbon-ion radiotherapy; HBV, hepatitis B virus; HCV, hepatitis C virus; mALBI grade, modified albumin–bilirubin grade; MASH, metabolic dysfunction-associated steatohepatitis; MVI, macrovascular invasion; PBC, primary biliary cholangitis; PIVKA-II, protein-induced vitamin K absence or antagonist-II; PLT, platelet count; PT, prothrombin time; RFA, radiofrequency ablation; TACE, transarterial chemoembolization; T-bil, total bilirubin.

**Table 3:** Characteristics of patients aged below 80 years undergoing hepatectomy and those receiving CIRT.

	CIRT (*n* = 19)	Hepatectomy (*n* = 13)	*p* Value
Age	Median: 71 (range: 56–79)	70 (58–77)	0.5613
Sex (male/female)	18/1	11/2	0.1900
Etiology (HBV/HCV/non-B, non-C/alcohol/MASH/PBC)	2/3/3/7/3/1	1/1/4/6/1/0	0.0756
mALBI grade (1/2a/2b/3)	12/4/3/0	10/2/1/0	0.0577
ALBI score	−2.648 (−3.415 to −1.313)	−2.786 (−3.186 to −1.800)	0.3497
BCLC stage (A/B/C)	16/0/3	8/4/1	0.6220
First treatment/pre-treatment (surgery/RFA/TACE/systemic therapy)	15/4	8/5	0.5903
Size (mm)	50.0 (40–110)	50.0 (40–130)	0.4290
Total number of intrahepatic tumors (1/2)	19/0	9/4	0.7422
MVI (−/+)	16/3	12/1	0.2048
Blood biochemistry (median [range])			
T-bil level (mg/dL)	0.70 (0.40–2.2)	0.70 (0.30–0.90)	0.0771
Alb level (g/dL)	4.0 (2.7–4.9)	4.1 (3.0–4.3)	0.5634
AST level (U/L)	29.0 (13–83)	24.0 (14–77)	0.4416
ALT level (U/L)	26.0 (10–95)	22.0 (13–107)	0.9393
PLT count (10^3^/µL)	162 (45–382)	190 (102–252)	0.8603
PT%	94.0 (65–112)	93.0 (68–123)	0.8019
AFP level (ng/mL)	4.0 (2.0–20,000)	4.0 (2.0–1273)	0.4414
PIVKA-II level (mAU/mL)	140.0 (13–1934)	82.0 (18.0–7719)	0.2008

AFP, alpha-fetoprotein; Alb, albumin; ALBI score, albumin–bilirubin score; ALT, alanine transaminase; AST, aspartate transaminase; BCLC, Barcelona Clinic Liver Cancer; CIRT, carbon-ion radiotherapy; HBV, hepatitis B virus; HCV, hepatitis C virus; mALBI grade, modified albumin–bilirubin grade; MASH, metabolic dysfunction-associated steatohepatitis; MVI, macrovascular invasion; PBC, primary biliary cholangitis; PIVKA-II, protein-induced vitamin K absence or antagonist-II; PLT, platelet count; PT, prothrombin time; RFA, radiofrequency ablation; TACE, transarterial chemoembolization; T-bil, total bilirubin.

**Table 4:** Characteristics of the CIRT patients aged <80 year and ≥80 year groups.

	CIRT, <80 Years (*n* = 19)	CIRT, ≥80 Years (*n* = 19)	*p* Value
Age	Median: 71 (range: 56–79)	85 (81–93)	<0.0001
Sex (male/female)	18/1	11/8	0.0188
Etiology (HBV/HCV/non-B, non-C/alcohol/MASH/PBC)	2/3/3/7/3/1	3/7/4/1/3/1	0.2655
mALBI grade (1/2a/2b/3)	12/4/3/0	8/5/5/1	0.4916
ALBI score	−2.648 (−3.415 to −1.313)	−2.563 (−3.075 to −1.744)	0.6024
BCLC stage (A/B/C)	16/0/3	14/3/2	0.2647
First treatment/pre-treatment (surgery/RFA/TACE/systemic therapy)	15/4	11/8	0.1496
Size (mm)	50.0 (40–110)	65.0 (40–105)	0.1510
Total number of intrahepatic tumors (1/2)	19/0	16/3	0.1870
MVI (−/+)	16/3	16/3	>0.9999
Blood biochemistry (median [range])			
T-bil level (mg/dL)	0.70 (0.40–2.2)	0.60 (0.40–1.4)	0.1052
Alb level (g/dL)	4.0 (2.7–4.9)	3.8 (2.7–4.5)	0.2510
AST level (U/L)	29.0 (13–83)	36.0 (12–222)	0.3784
ALT level (U/L)	26.0 (10–95)	32.0 (5.0–135)	0.9815
PLT count (10^3^/µL)	162 (45–382)	194 (70–371)	0.4927
PT%	94.0 (65–112)	94.0 (81–114)	0.4667
AFP level (ng/mL)	4.0 (2.0–20,000)	6.7 (2.0–20,000)	0.3463
PIVKA-II level (mAU/mL)	140.0 (13–1934)	785.0 (15.0–81,886)	0.0542

AFP, alpha-fetoprotein; Alb, albumin; ALBI score, albumin–bilirubin score; ALT, alanine transaminase; AST, aspartate transaminase; BCLC, Barcelona Clinic Liver Cancer; CIRT, carbon-ion radiotherapy; HBV, hepatitis B virus; HCV, hepatitis C virus; mALBI grade, modified albumin–bilirubin grade; MASH, metabolic dysfunction-associated steatohepatitis; MVI, macrovascular invasion; PBC, primary biliary cholangitis; PIVKA-II, protein-induced vitamin K absence or antagonist-II; PLT, platelet count; PT, prothrombin time; RFA, radiofrequency ablation; TACE, transarterial chemoembolization; T-bil, total bilirubin.

**Table 5:** Adverse events after CIRT.

		Grade 1	Grade 2	Grade 3	Grade 4	Grade 5	Time to Onset (Month) Median (Range)
Liver dysfunction	*n* = 13 (34.2%)	11	2	0	0	0	2.2 (1.0–6.0)
Fatigue	*n* = 12 (31.6%)	12	0	0	0	0	2.0 (0.5–5.0)
Skin redness/dermatitis	*n* = 11 (28.9%)	9	2	0	0	0	1.5 (1.0–6.0)
Anorexia	*n* = 6 (15.8%)	6	0	0	0	0	1.5 (0.5–3.0)
Localized pleural effusion	*n* = 3 (7.9%)	3	0	0	0	0	4.5 (3.0–6.0)
Itchy skin	*n* = 3 (7.9%)	3	0	0	0	0	2.5 (1.0–8.0)
Rib fracture/myositis	*n* = 3 (7.9%)	1	2	0	0	0	6.0 (3.0–15.0)
Nausea	*n* = 1 (2.6%)	1	0	0	0	0	3.0 (3.0–3.0)
Biliary stricture	*n* = 1 (2.6%)	0	0	1	0	0	5.0 (5.0–5.0)

## Data Availability

The data supporting the findings of this study are available from the corresponding author (H.H.) upon reasonable request.

## Supplementary Materials

The following supporting information can be downloaded at https://www.mdpi.com/article/10.3390/jcm14165678/s1, Figure S1: Intrahepatic recurrence in patients who underwent CIRT or hepatectomy for HCC measuring ≥4 cm. Recurrence was categorized as local or intrahepatic distant recurrence, and the rates were compared between the groups. CIRT, carbon-ion radiotherapy; HCC, hepatocellular carcinoma; Figure S2: Flowchart of selecting patients who underwent CIRT or hepatectomy for HCC measuring ≥4 cm. BSC, best supportive care; TAE, transarterial embolization; Figure S3: Comparison of disease control rates between the CIRT and hepatectomy groups using matching. Table S1: Previous clinical study results in patients with HCC treated with CIRT.

## Abbreviations

ALBI score	albumin–bilirubin score
BCLC	Barcelona Clinic Liver Cancer
CIRT	carbon-ion radiotherapy
CR	complete response
CT	computed tomography
CTV	clinical target volume
GTV	gross tumor volume
HCC	hepatocellular carcinoma
ITV	internal target volume
LEN	lenvatinib
mALBI grade	modified albumin–bilirubin grade
MASH	metabolic dysfunction-associated steatohepatitis
MRI	magnetic resonance imaging
OAR	organs at risk
PBC	primary biliary cholangitis
PD	progressive disease
PFS	progression-free survival
PR	partial response
RFA	radiofrequency ablation
SD	stable disease
TACE	transarterial chemoembolization
