# Supplementary material for: Treatment Outcomes in Patients Receiving Carbon-Ion Radiotherapy Versus Hepatectomy for Hepatocellular Carcinoma (≥4 cm): A Retrospective Study in Japan
Source: J Clin Med. 2025 Aug 11;14(16):5678. doi: 10.3390/jcm14165678 (PMC12386234; doi:10.3390/jcm14165678)
Supplement: Supplementary file 1 [file jcm-14-05678-s001.zip › jcm-3770846-supplementary.pptx]

## Slide 1
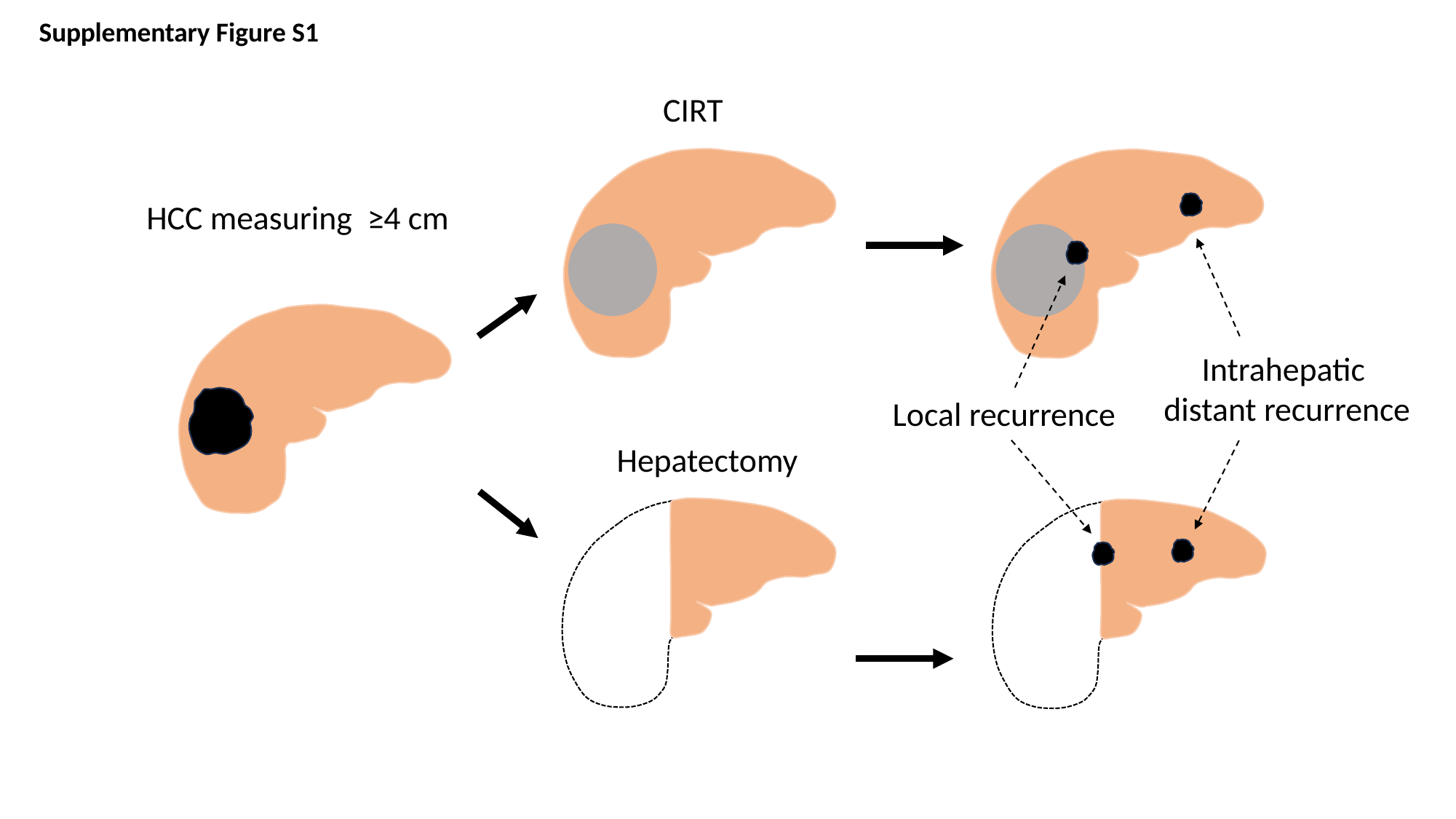

Supplementary Figure S1
CIRT
HCC measuring ≥4 cm
Intrahepatic
distant recurrence
Local recurrence
Hepatectomy

## Slide 2
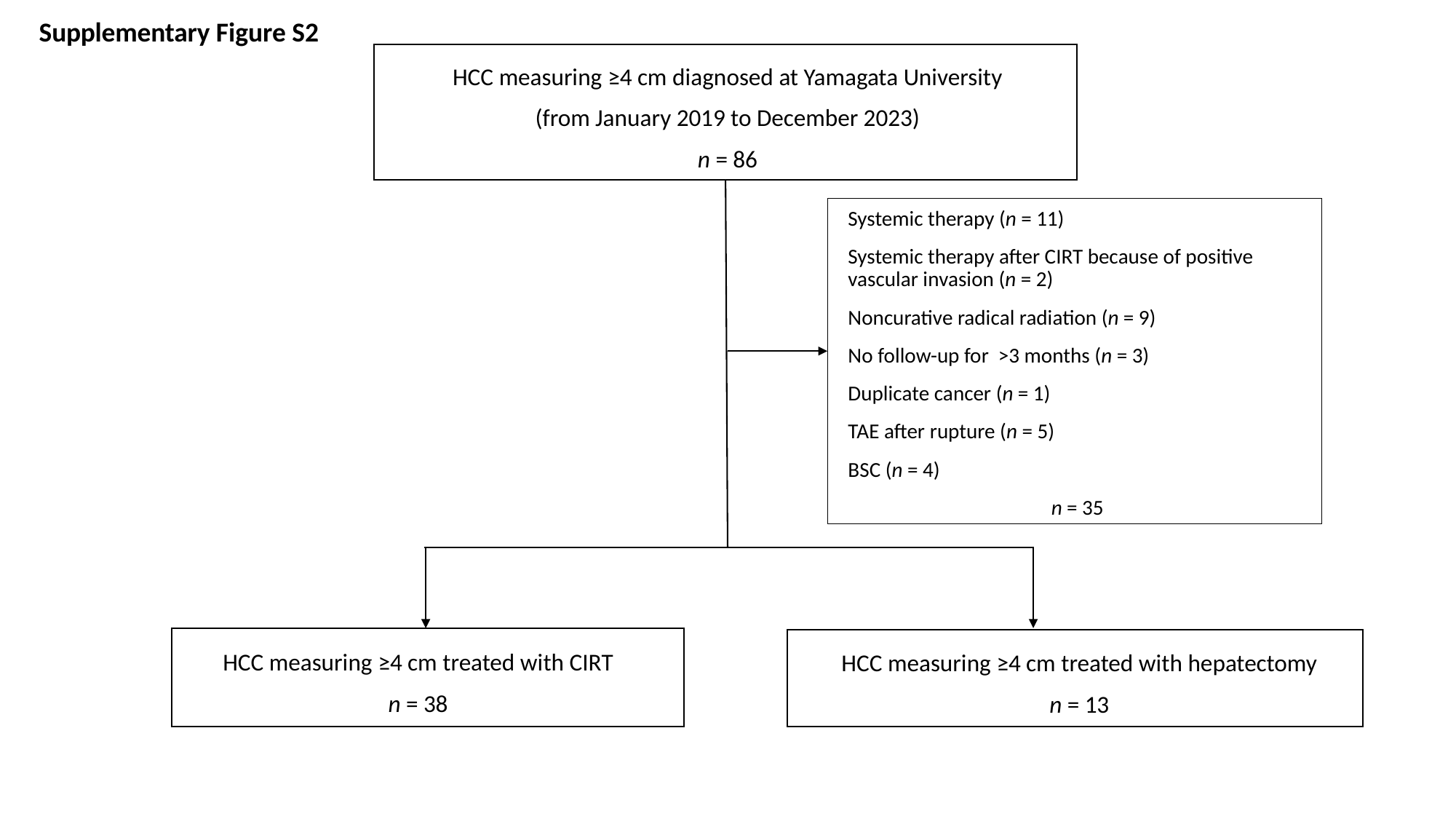

Supplementary Figure S2
HCC measuring ≥4 cm diagnosed at Yamagata University
(from January 2019 to December 2023)
n = 86
Systemic therapy (n = 11)
Systemic therapy after CIRT because of positive vascular invasion (n = 2)
Noncurative radical radiation (n = 9)
No follow-up for >3 months (n = 3)
Duplicate cancer (n = 1)
TAE after rupture (n = 5)
BSC (n = 4)
 n = 35
HCC measuring ≥4 cm treated with CIRT
n = 38
HCC measuring ≥4 cm treated with hepatectomy
n = 13

## Slide 3
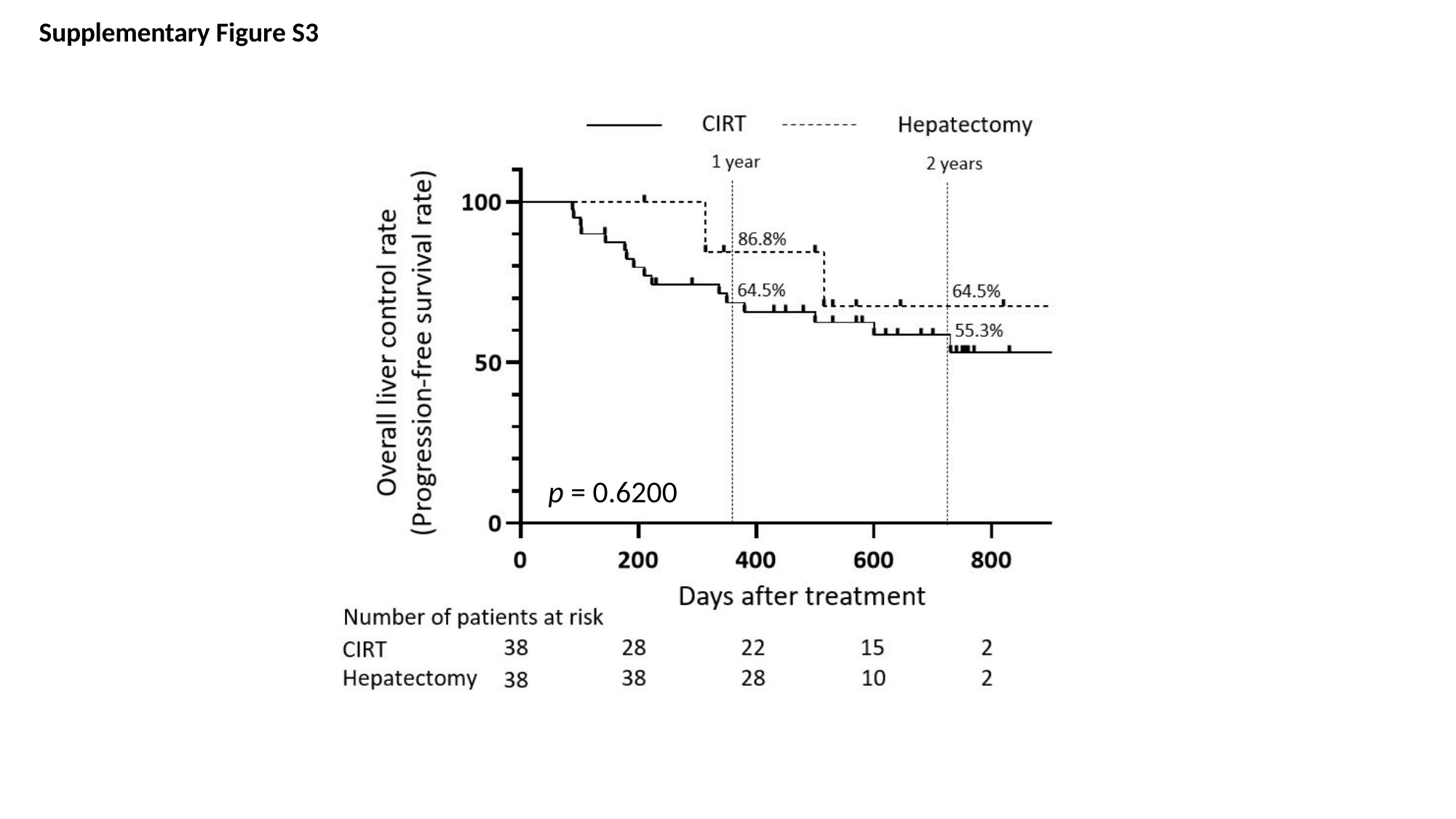

Supplementary Figure S3
p = 0.6200

## Slide 4
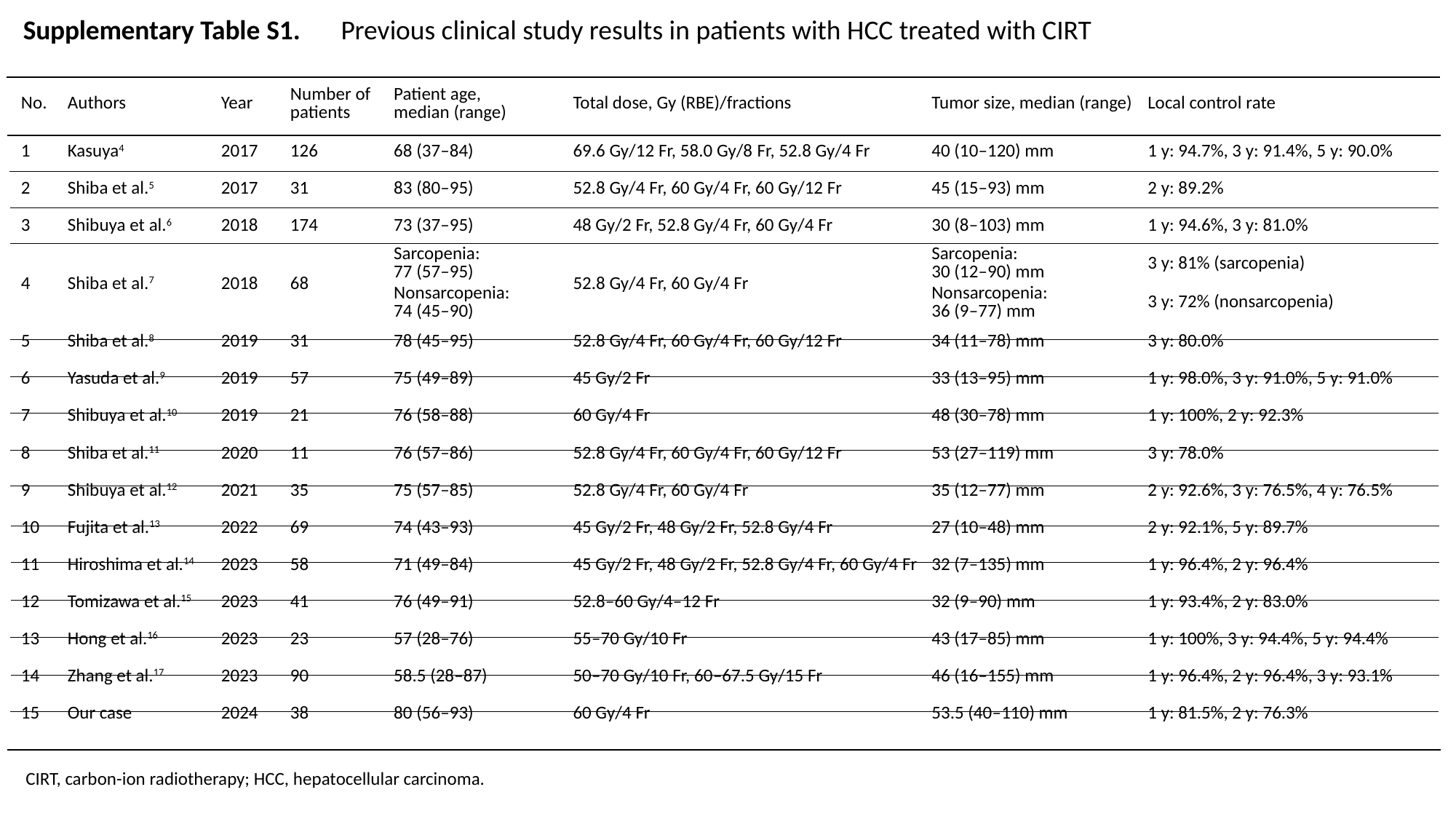

Supplementary Table S1.　Previous clinical study results in patients with HCC treated with CIRT
| No. | Authors | Year | Number of patients | Patient age, median (range) | Total dose, Gy (RBE)/fractions | Tumor size, median (range) | Local control rate |
| --- | --- | --- | --- | --- | --- | --- | --- |
| 1 | Kasuya4 | 2017 | 126 | 68 (37–84) | 69.6 Gy/12 Fr, 58.0 Gy/8 Fr, 52.8 Gy/4 Fr | 40 (10–120) mm | 1 y: 94.7%, 3 y: 91.4%, 5 y: 90.0% |
| 2 | Shiba et al.5 | 2017 | 31 | 83 (80–95) | 52.8 Gy/4 Fr, 60 Gy/4 Fr, 60 Gy/12 Fr | 45 (15–93) mm | 2 y: 89.2% |
| 3 | Shibuya et al.6 | 2018 | 174 | 73 (37–95) | 48 Gy/2 Fr, 52.8 Gy/4 Fr, 60 Gy/4 Fr | 30 (8–103) mm | 1 y: 94.6%, 3 y: 81.0% |
| 4 | Shiba et al.7 | 2018 | 68 | Sarcopenia: 77 (57–95) | 52.8 Gy/4 Fr, 60 Gy/4 Fr | Sarcopenia: 30 (12–90) mm | 3 y: 81% (sarcopenia) |
| | | | | | | Nonsarcopenia: 36 (9–77) mm | 3 y: 72% (nonsarcopenia) |
| | | | | Nonsarcopenia: 74 (45–90) | | | |
| 5 | Shiba et al.8 | 2019 | 31 | 78 (45–95) | 52.8 Gy/4 Fr, 60 Gy/4 Fr, 60 Gy/12 Fr | 34 (11–78) mm | 3 y: 80.0% |
| 6 | Yasuda et al.9 | 2019 | 57 | 75 (49–89) | 45 Gy/2 Fr | 33 (13–95) mm | 1 y: 98.0%, 3 y: 91.0%, 5 y: 91.0% |
| 7 | Shibuya et al.10 | 2019 | 21 | 76 (58–88) | 60 Gy/4 Fr | 48 (30–78) mm | 1 y: 100%, 2 y: 92.3% |
| 8 | Shiba et al.11 | 2020 | 11 | 76 (57–86) | 52.8 Gy/4 Fr, 60 Gy/4 Fr, 60 Gy/12 Fr | 53 (27–119) mm | 3 y: 78.0% |
| 9 | Shibuya et al.12 | 2021 | 35 | 75 (57–85) | 52.8 Gy/4 Fr, 60 Gy/4 Fr | 35 (12–77) mm | 2 y: 92.6%, 3 y: 76.5%, 4 y: 76.5% |
| 10 | Fujita et al.13 | 2022 | 69 | 74 (43–93) | 45 Gy/2 Fr, 48 Gy/2 Fr, 52.8 Gy/4 Fr | 27 (10–48) mm | 2 y: 92.1%, 5 y: 89.7% |
| 11 | Hiroshima et al.14 | 2023 | 58 | 71 (49–84) | 45 Gy/2 Fr, 48 Gy/2 Fr, 52.8 Gy/4 Fr, 60 Gy/4 Fr | 32 (7–135) mm | 1 y: 96.4%, 2 y: 96.4% |
| 12 | Tomizawa et al.15 | 2023 | 41 | 76 (49–91) | 52.8–60 Gy/4–12 Fr | 32 (9–90) mm | 1 y: 93.4%, 2 y: 83.0% |
| 13 | Hong et al.16 | 2023 | 23 | 57 (28–76) | 55–70 Gy/10 Fr | 43 (17–85) mm | 1 y: 100%, 3 y: 94.4%, 5 y: 94.4% |
| 14 | Zhang et al.17 | 2023 | 90 | 58.5 (28–87) | 50–70 Gy/10 Fr, 60–67.5 Gy/15 Fr | 46 (16–155) mm | 1 y: 96.4%, 2 y: 96.4%, 3 y: 93.1% |
| 15 | Our case | 2024 | 38 | 80 (56–93) | 60 Gy/4 Fr | 53.5 (40–110) mm | 1 y: 81.5%, 2 y: 76.3% |
CIRT, carbon-ion radiotherapy; HCC, hepatocellular carcinoma.
